# Significance of day-to-day glucose variability in patients after acute coronary syndrome

**DOI:** 10.1186/s12872-021-02303-z

**Published:** 2021-10-10

**Authors:** Machiko Miyoshi, Hiroyasu Uzui, Tomohiro Shimizu, Takayoshi Aiki, Yuichiro Shiomi, Minoru Nodera, Hiroyuki Ikeda, Naoto Tama, Kanae Hasegawa, Tetsuji Morishita, Kentaro Ishida, Shinsuke Miyazaki, Hiroshi Tada

**Affiliations:** Department of Cardiovascular Medicine, Faculty of Medical Sciences, University of Fukui, 23-3 Shimoaizuki, Matsuoka Eiheiji-Cho, Fukui, 910-1193 Japan

**Keywords:** Day-to-day glucose variability, Acute coronary syndrome, Mean of daily differences

## Abstract

**Background:**

Several studies have recently addressed the importance of glycemic variability (GV) in patients with acute coronary syndrome (ACS). Although daily GV measures, such as mean amplitude of glycemic excursions, are established predictors of poor prognosis in patients with ACS, the clinical significance of day-to-day GV remains to be fully elucidated. We therefore monitored day-to-day GV in patients with ACS to examine its significance.

**Methods:**

In 25 patients with ACS, glucose levels were monitored for 14 days using a flash continuous glucose monitoring system. Mean of daily differences (MODD) was calculated as a marker of day-to-day GV. N-terminal pro-brain natriuretic peptide (NT-proBNP) was evaluated within 4 days after hospitalization. Cardiac function (left ventricular end-diastolic volume, left ventricular ejection fraction, stroke volume) was assessed by echocardiography at 3–5 days after admission and at 10–12 months after the disease onset.

**Results:**

Of the 25 patients, 8 (32%) were diagnosed with diabetes, and continuous glucose monitoring (CGM)-based MODD was high (16.6 to 42.3) in 17 patients (68%). Although MODD did not correlate with max creatine kinase (CK), there was a positive correlation between J-index, high blood glucose index, and NT-proBNP (*r* = 0.83, *p* < 0.001; *r* = 0.85, *p* < 0.001; *r* = 0.41, *p* = 0.042, respectively).

**Conclusions:**

In patients with ACS, MODD was associated with elevated NT-proBNP. Future studies should investigate whether day-to-day GV in ACS patients can predict adverse clinical events such as heart failure.

## Background

Several studies have recently addressed the importance of glycemic variability (GV) in patients with acute coronary syndrome (ACS) [1–4]. Continuous glucose monitoring (CGM) systems are an emerging technology that can continuously measure glucose levels, thereby enabling evaluation of GV [5, 6]. Su et al. reported that monitoring GV using CGM can predict mortality and major adverse cardiovascular events in elderly patients after acute myocardial infarction [7]. They also reported that high GV at admission may be closely correlated with in-hospital poor outcomes in diabetes mellitus (DM) patients with non-ST segment elevation ACS following percutaneous coronary intervention (PCI) [8]. Furthermore, using CGM to monitor daily GV parameters such as mean amplitude of glycemic excursions (MAGE) is a predictor of poor prognosis in patients with ACS without severe DM [9]. In-hospital daily GV in the stable phase of ST-elevation myocardial infarction predicts left ventricular remodeling, as determined by cardiac magnetic resonance imaging [10].

However, the clinical significance of day-to-day GV in patients with ACS remains to be fully elucidated. Therefore, we monitored day-to-day GV in patients with ACS to examine its clinical significance.

## Methods

### Subjects

We enrolled 25 patients who were admitted to the University of Fukui Hospital with ACS between September 2017 and March 2018. Patients with hemodynamic instability, such as those with complications of infection, catecholamine or sedative use, ventilator management, or ventricular arrhythmia, were excluded because of concerns about the effects of infection, drugs, and severe stress on blood glucose levels. Patients on insulin were also excluded because of the possible effect on blood glucose variability.

Smoking is defined as any smoking in the past year. Diabetes was defined based on one or more of the following: self-reported, use of diabetes medications, fasting plasma glucose ≥ 126 mg/dl, or hemoglobin A1c (National Glycohemoglobin Standardization Program) ≥ 6.5%. Clinical histories of the patients were obtained from interviews with patients.

### Study protocol

After the purpose and methods of the study were explained to patients, they provided written informed consent for participation in the study. In this study, continuous glucose levels were monitored using a flash glucose monitoring (FGM) system (Free-Style Libre™ or Free-Style Libre Pro, Abbott™, UK). All patients were implanted with sensors of Free-Style Libre™ or Free-Style Libre Pro™ in their left upper arm within 3–5 days after hospitalization. Glucose levels were recorded using the FGM system for up to 14 days, excluding the first 2 days after sensor implantation because of the risk of errors due to inflammatory reactions, which could produce unstable glucose data. After analysis of the CGM data, MAGE, MODD, ADRR (average daily risk range, mg/dl), J-index, M-value (mg/dl), LBGI (low blood glucose index), and HBGI (high blood glucose index) were calculated using EasyGV^©^ software. The formulas for each parameter are based on Nathan et al. [11] Chen et al. reported the MODD of non-diabetic patients is 16.0 ± 5.4 mg/dl [12]. Therefore, a MODD value of 16.0 or higher was chosen as the abnormal value. MODD was calculated by $\frac{\sum_{t={t}_{1}}^{{t}_{k}}|{G}_{t}-{G}_{t-1440}|}{k}$ (*k* = number of observations with an observation 24 h ago, *G* = glucose measured, *t* = time). To assess cardiac function, patients underwent echocardiography at 3–5 days after admission and at 10–12 months after the disease onset. The patients' diet consisted of a cardiac diet of 30 kcal/kg in ideal body weight in three portions. They did not receive any intravenous catecholamines or antibiotics. This investigation conformed to the principles outlined in the 1975 Declaration of Helsinki and later amendments. This study was approved by the Research Ethics Committee of University of Fukui and informed consent from all patients were obtained, and the follow-up results were registered in the Universal Hospital Medical Information Network Clinical Trials Registry (UMIN 000023837, 30/08/2016).

### Measurements of blood samples

Within 4 days after hospitalization, following overnight fasting, blood samples were collected from the peripheral vein of each patient and kept on ice. Plasma was collected with EDTA-2Na as an anti-coagulant, and serum samples were separated by centrifugation within 30 min. Blood parameters were determined using standard methods.

### Statistical analysis

All statistical analyses were performed using Statcel2 software (OMS Publishing Inc., Saitama, Japan) and Excel2019 (Microsoft Corporation). Data are presented as frequencies and percentages for categorical variables and mean ± SD for continuously distributed variables. Differences between categorical variables were assessed using the χ^2^ test. Correlations between continuous variables were determined using Pearson’s correlation coefficient test. A *p* value of < 0.05 was considered statistically significant.

## Results

### Patient characteristics

The characteristics of all patients are listed in Table 1. The mean age was 69.7 years, and 84% of patients were male. The study included 25 patients (14 patients with ST-elevated acute myocardial infarction, 6 patients with non-ST-elevated acute myocardial infarction, and 5 patients with unstable angina). In the study, 72% had a medical history of hypertension, and 24% had hypercholesterolemia. DM was observed in 8 patients (32%), and 7 patients (28%) were taking one or more antidiabetes medications. The mean value of peak CK was 1310.1 ± 1293.0 U/l, and NT-pro BNP was 2032.0 ± 2063.3 pg/ml (Table 1).

**Table 1:** Patient characteristics

Characteristic	N = 25
Age (years)	69.7 ± 10.9
Males (%)	21 (84.0)
Risk factors	
DM (%)	8 (32)
Smoking (%)	9 (36)
BMI (kg/m^2^)	23.1 ± 3.31
HT (%)	18 (72)
Previous CAD (%)	2 (8)
CKD (%)	11 (44)
DLP (%)	6 (24)
Medications	
Antiplatelet drug (%)	5 (20)
Beta-blocker (%)	1 (4)
ACE-I/ARB (%)	11(44)
CCB (%)	10 (40)
Statin (%)	6 (24)
Anti-hyperuricemic drug (%)	3 (12)
Oral antidiabetes drug (%)	7 (28)
Blood tests	
TG, mg/dl	103.7 ± 54.0
LDL, mg/dl	98.32 ± 36.3
HDL, mg/dl	44.6 ± 9.7
1-5 AG, μg/ml	16.7 ± 8.6
apoA-I, mg/dl	105.2 ± 16.5
apoB, mg/dl	75.3 ± 23.1
apoE, mg/dl	2.8 ± 0.85
DHLA, μg/ml	33.7 ± 12.1
AA, μg/ml	166.6 ± 41.8
EPA, μg/ml	58.5 ± 42.4
DHA, μg/ml	119.3 ± 48.3
EPA/AA	0.35 ± 0.22
NT-proBNP, pg/ml	2032.0 ± 2063.3
MDA-LDL, U/l	80.3 ± 29.6
peakCK, U/l	1310.1 ± 1293.0
HbA1c, %	6.23 ± 0.70
Type of ACS	
STEMI (%)	14 (56)
Non-STEMI (%)	6 (24)
UAP (%)	5 (20)

*AA* arachidonic acid, *ACE-I* angiotensin-converting-enzyme, *1-5 AG* 1-5 anhydroglucitol, *apoA-I* apolipoprotein A-I, *apoB* apolipoprotein B, *apoE* apolipoprotein E, *ARB* angiotensin II receptor blocker, *BMI* body mass index, *CAD* coronary artery disease, *CCB* calcium channel blocker, *CK* creatine kinase, *CKD* chronic kidney disease, *DHA* docosahexaenoic acid, *DHLA* dihydrogammalinolenic acid, *DLP* dyslipidemia, *DM* diabetes mellitus, *EPA* eicosapentaenoic acid, *HbA1c* hemoglobin A1c, *HDL* high-density lipoprotein, *HT* hypertension, *LDL* low-density lipoprotein, *MDA-LDL* malondialdehyde-modified low-density lipoprotein, *NT-proBNP* N-terminal pro-brain natriuretic peptide, *STEMI* ST segment-elevated myocardial infarction, *TG* triglyceride, *UAP* unstable angina pectoris

### Prevalence of MODD and DM

**Fig. 1:**
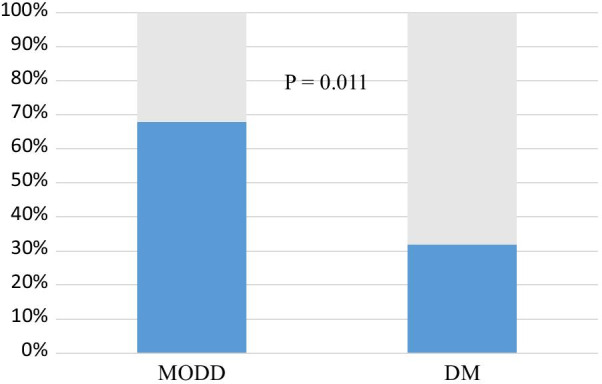
Prevalence of MODD and DM. Detection rate of MODD abnormalities was significantly higher than the diagnosed rate of DM (32% vs. 96%, *p* < 0.001). *DM* diabetes mellitus, *MODD* mean of daily differences

On admission, only 8 patients (32%) were diagnosed with diabetes. On the other hand, as many as 17 of 25 patients (68%) had abnormally high MODD values. The rate of abnormal MODD was significantly higher than the diagnosed rate of DM (32% vs. 68%, *p* = 0.011) (Fig. 1).

### Correlation of MODD and NT-pro BNP

MODD was correlated with MAGE, J-index, and M-value (mg/dl), which are indicators of GV. MODD was also correlated with LBGI, HBGI, and ADRR, which is another indicator of day-to-day GV. There was no correlation between MODD and lipid-related coronary risk factors such as LDL, apoB, and EPA/AA (Table 2). There was no correlation between clinical parameters such as age, renal function, and echocardiography measurements and markers of blood glucose variability, including MODD.

**Table 2:** Single correlation analysis between MODD and clinical characteristics, other indices of blood glucose fluctuation

	*R*	*p*
MAGE (mg/dl)	0.85	< 0.001
ADRR (mg/dl)	0.86	< 0.001
M-value (mg/dl)	− 0.08	0.7
J-index	0.83	< 0.001
LBGI	− 0.24	0.24
HBGI	0.85	< 0.001
HbA1c (%)	0.47	0.019
1-5 AG (μg/ml)	− 0.22	0.3
TG (mg/dl)	− 0.19	0.35
LDL (mg/dl)	− 0.0096	0.96
HDL (mg/dl)	− 0.19	0.37
apoA-I (mg/dl)	− 0.38	0.063
apoB (mg/dl)	0.054	0.8
apoE (mg/dl)	− 0.011	0.96
DHLA (μg/ml)	− 0.23	0.27
AA (μg/ml)	0.15	0.46
EPA (μg/ml)	− 0.0082	0.97
DHA (μg/ml)	− 0.12	0.56
EPA/AA	− 0.072	0.73
MDA-LDL (U/l)	− 0.023	0.91
NT-pro BNP (pg/ml)	0.41	0.042
maxCK (U/l)	0.25	0.23
eGFR (ml/min/1.73 m^2^)	− 0.26	0.11
Age	0.22	0.85
Acute phase LVEF (%)	− 0.21	0.30
Acute phase EDV (ml)	− 0.25	0.23
Acute phase SV (ml)	− 0.39	0.06
ΔLVEF (%)	− 0.13	0.54
ΔEDV (ml)	− 0.38	0.06
ΔSV (ml)	− 0.38	0.06

Acute phase = 3–5 days after admission; chronic phase = 10–12 months after the disease onset; ΔEF = chronic phase EF–acute phase EF; ΔEDV = chronic phase EDV–acute phase EDV; ΔSV = chronic phase SV–acute phase SV

*ADRR* average daily risk range, *AA* arachidonic acid, *apoA-I* apolipoprotein A-I, *apoB* apolipoprotein B, *apoE* apolipoprotein E, *CK* creatine kinase, *DHA* docosahexaenoic acid, *DHLA* dihydrogammalinolenic acid, *EDV* end-systolic volume, *eGFR* estimated glomerular filtration rate, *EPA* eicosapentaenoic acid, *HbA1c* hemoglobin A1c, *HBGI* high blood glucose index, *HDL* high-density lipoprotein, *LBGI* low blood glucose index, *LDL* low-density lipoprotein, *LVEF* left ventricular ejection fraction, *MAGE* mean amplitude of glycemic excursions, *MDA-LDL* malondialdehyde-modified low-density lipoprotein, *NT-proBNP* N-terminal pro-brain natriuretic peptide, *SV* stroke volume, *TG* triglyceride

**Fig. 2:**
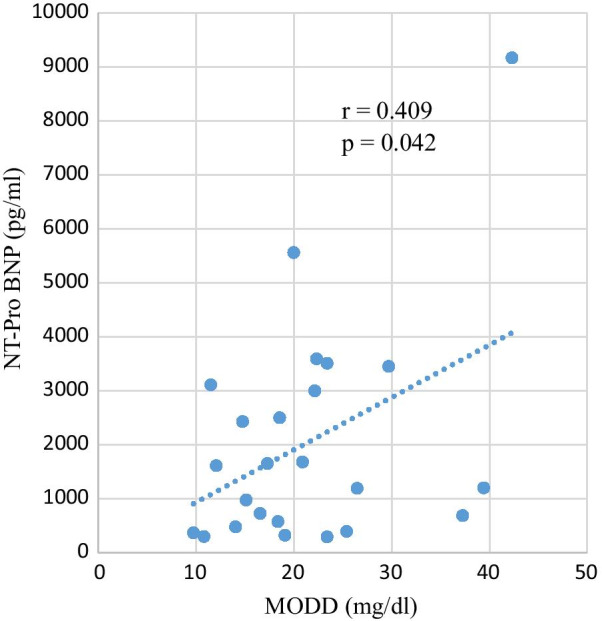
Correlation between MODD and NT-proBNP. A positive correlation was found between MODD and NT-proBNP (*r* = 0.409, *p* = 0.042). *MODD* mean of daily differences, *NT-proBNP* N-terminal pro-brain natriuretic peptide

A positive correlation was found between MODD and NT-pro BNP (*r* = 0.409, *p* = 0.042), although MODD did not correlate with max CK (Table 2, Fig. 2). HBGI was correlated with NT-pro BNP (r = 0.46, *p* = 0.019) as well as MODD.

Using the cut off value of 400 pg/ml, the reference value for heart failure in NT-proBNP, 19 of 25 patients were abnormal. The mean MODD of the group with high NT-proBNP (≥ 400 pg/ml) was 22.2 and the percentage of abnormalities was 73.7%, while the mean MODD of the group with low NT-proBNP was 17.6 and the percentage of abnormalities was 60%, which was not a significant difference. The high MODD group (≥ 16 mg/dl) had a mean NT-proBNP of 2323 pg/ml, 82.4% of abnormalities, while the MODD normal group had a mean NT-proBNP of 1324 pg/ml, 71.4% of abnormalities, also not significantly different.

## Discussion

The main findings of the present study were as follows. First, among 25 patients with ACS, the prevalence of abnormal MODD was high (68%) compared to the prevalence of DM (38%). Second, there was an association between MODD, a parameter of day-to-day GV, and NT-pro BNP, a parameter of poor prognosis.

In the present study, 8 patients (32%) were diagnosed with diabetes at or before admission to the hospital. In contrast, MODD assessed by CGM was found to be over the normal level (> 16.0) in 17 (68%) patients. These results suggest that CGM may provide indications of diabetes and glucose intolerance in some patients who were considered normal using previous diagnostic methods. Previous reports have shown that when oral glucose tolerance testing (OGTT) was performed on patients admitted with ACS, 24% were diagnosed as diabetic, 38% as impaired glucose tolerance, and the remaining 38% as normal [13, 14]. Day-to-day assessment of blood glucose using CGM could identify patients with blood glucose variations beyond daily glucose variability.

In this study, we found that many patients with ACS had GV. Previous reports have suggested that high GV is an important factor in coronary plaque vulnerability [15, 16], and our results may also suggest an association between GV and plaque vulnerability.

Although day-to-day GV in the acute phase may differ from day-to-day variability in regular outpatient care [17], it was suggested that incorporating CGM into routine diabetes care and measuring MODD may help to differentiate patients at high risk for ACS who are being impacted by GV.

We also examined the association between various markers of GV calculated from CGM and other indices. HBGI was also abnormal in 4 of 25 patients and correlated with BNP. Despite the lack of correlation between peak CK and MODD, MODD was found to be associated with NT-pro BNP. MODD was not correlated with CK, which reflects the degree of myocardial infarction. In addition, there was no correlation between infarct size and MODD. On the other hand, the fact that MODD associated with NT-pro BNP, a marker of poor prognosis, suggests that patients with abnormal MODD have some factors associated with poor prognosis in addition to the myocardial infarction that led to hospitalization. These results are consistent with previous reports that daily and day-to-day GV increase oxidative stress and inflammation, which cause myocardial damage [18–21]. Recent investigations suggest that α-glucosidase inhibitor and glucagon-like peptide-1 analogue attenuate GV and inhibit oxidative injury [22, 23]. Therefore, we could potentially improve prognosis using these drugs.

### Clinical implications

There have been no studies comparing MODD with traditional BNP or myocardial infarction. The significance of measuring MODD in patients with ACS is that the measurement can predict poor cardiac prognosis beyond those expected based on infarct volume and may serve as a marker for more-intensive anti-cardiac therapy. In addition, CGM in DM routine practice may help in risk stratification of vulnerable patients with ACS.

## Limitations

Although some of the patients in this study had high HbA1c levels, these patients may have had less blood glucose variability and been less likely to have MODD abnormalities and therefore should have been omitted from the study, we included them due to the limited number of cases. Therefore, the correlation between MODD and NT-pro BNP in this study may be weak. In addition, OGTT was not used to diagnose diabetes at hospitalization. This may have resulted in a lower rate of diabetes diagnosis. However, the rate of elevated MODD was higher than the number of diagnoses of DM comorbidities and new DM cases on admission for ACS previously reported. NT-pro BNP can be elevated by chronic kidney disease, and it was present in 44% of our patient group. However, there was no correlation between MODD and eGFR values. In this study, all patients with ACS were undergoing inpatient care, and we did not score heart failure symptoms nor evaluate the association between symptoms and NT-pro BNP. All of the patients included in this study had undergone PCI, and although this invasive procedure may affect GV, they all had this procedure in common and did not undergo other invasive procedures such as the use of devices that affect hemodynamics. The blood glucose variability observed in this study may include not only the existing blood glucose variability caused by DM, but also the effect of the stress response of ACS. Therefore, the stress response may have influenced the result of the high MODD abnormality rate relative to the DM patient ratio. However, stress-induced hyperglycemia itself is also said to be a poor prognostic factor [24], and the association between MODD and NT-pro BNP in the present study seems to be consistent with previous reports.

## Conclusions

In conclusion, CGM estimations of MODD, a parameter of day-to-day GV, in ACS patients were abnormal at a high rate. In patients with ACS, MODD was associated with elevated NT-proBNP. Future studies should investigate whether day-to-day GV in ACS patients can predict adverse clinical events such as heart failure.

## Data Availability

The datasets generated during and analyzed during the current study are not publicly available due to the participants in this study not consenting to the release of their data, but are available from the corresponding author on reasonable request.

## Abbreviations

GV: Glycemic variability
ACS: Acute coronary syndrome
MODD: Mean of daily differences
NT-pro BNP: N-terminal pro-brain natriuretic peptide
CGM: Continuous glucose monitoring
CK: Creatine kinase
DM: Diabetes mellitus
PCI: Percutaneous coronary intervention
MAGE: Mean amplitude of glycemic excursions
FGM: Flash glucose monitoring
ADRR: Average daily risk range
LBGI: Low blood glucose index
HBGI: High blood glucose index
OGTT: Oral glucose tolerance testing
